# Experimental and modeling investigation of organic modified montmorillonite with octyl quaternary ammonium salt

**DOI:** 10.1038/s41598-022-18253-1

**Published:** 2022-08-22

**Authors:** Hongyan Liu, Chengxin Guo, Yingna Cui, Jingmei Yin, Shenmin Li

**Affiliations:** 1 College of Environment and Chemical Engineering, Dalian University, Dalian, 116622 China; 2 Liaoning Key Laboratory of Bioorganic Chemistry, Dalian University, Dalian, 116622 China

**Keywords:** Nanoscale materials, Theory and computation

## Abstract

The sodium montmorillonite was organic modified with three kinds of quaternary ammonium salts containing 1 to 3 octyl chains, and then the organic montmorillonite was studied by FT-IR, XRD, and TG characterization as well as Monte Carlo simulations, to explore the influence of the number of octyl chains and the loading of intercalated cations on the basal spacing (d_001_) of the modified montmorillonite complexes. According to the distribution of intercalated quaternary ammonium cations and the energy change of the montmorillonite complexes, a reasonable explanation was given for the enlargement of the interlayer space. The results of experimental characterization and Monte Carlo simulations show that all the three intercalation agents can enlarge the interlayer space of montmorillonite complexes. The more the number of octyl chains in the salt, the more significant expanding effect on the interlayer space. The three intercalation cations exhibited a distribution arranged from mono-layered to multi-layered structure as the loading of intercalated cations increases.

## Introduction

Montmorillonite (Mt) is one of the most common clay minerals in nanocomposites. Due to its unique structure, it has good adsorption and thermal stability, which has attracted widespread attention from chemical and material scientists^1–7^. Alkyl quaternary ammonium salts (QAS) are often used as intercalating agents in the organic modification of Mt^8–11^. Luo and Zhou et al. organic modified Mt by ion exchange reaction, which allowed the QAS cations to enter the interlayer of Mt, effectively expanding the interlayer space, making it from hydrophilic to hydrophobic^12,13^, therefore, it has been applied successfully to the adsorption of pollutants and toxic gases^14–17^. Through FT-IR, XRD, TG, SEM experimental characterization, Yang et al. found that the organic montmorillonite(OMt) is closely related to the experimental QAS concentration, and speculated the QAS loading as well as the possible arrangement of those intercalating cations between the Mt layers^18,19^. Qian et al. modified sodium montmorillonite (Na-Mt) by a series of hexadecylmethyl ammonium salts with various number of alkyl chains of 1C16, 2C16 and 3C16, they found that the interlayer space increases to 2.04, 4.01 and 5.07 nm, respectively. The data indicate that the interlayer space increases dramatically with the increase of the number of alkyl chains. Also, They found the effect of the number of alkyl chains on the interlayer space is more important than the length of alkyl chain^20^.

Due to the complexity of the intercalation structure, the understanding of the structure and physical and chemical properties of organic modified montmorillonite with QAS needs to be further explored. Previous research reports on OMt are more common in short-chained or long-chained alkyl QAS, and few reports on the QAS containing C4 to C10 alkyl chains. In this paper, three kinds of QAS containing octyl chains (octyl trimethyl ammonium chloride (OTAC), dioctyl dimethyl ammonium chloride (BDAC), and trioctyl methyl ammonium chloride (TOMAC) were used to obtain the OMt complexes of OTA-Mt, BDA-Mt, and TOMA-Mt, respectively. FT-IR, XRD, TG, etc. were used to explore the influence of the number of octyl chains and the loading of QAS on the interlayer space of those OMt. On the other hand, as conventional characterization methods are difficult to accurately give molecular-level information^21,22^, based on TG characterization data, MC molecular simulations were used to investigate the distribution of intercalation QAS in the Mt interlayer at equilibrium. Through the mutual verification of experimental and simulation results, some new features are revealed.

## Experimental and simulation details

### Materials

OTAC, TOMAC were purchased from Aladdin Reagent (Shanghai) Co., BDAC, were purchased from Shanghai Yuanye Biotechnology Co. The raw clay, sodium montmorillonite (Na-Mt), with a cation exchange capacity (CEC) of 90 mmol/100 g, was provided by Henan Gongyi Hengxin Filter Material Factory. All reagents were used as received. Deionized water was used throughout the experiment.

### Preparation of QAS modified Mt

1.5 g of Mt was initially dispersed in a three-necked flask with 150 mL of deionized water for 1 h at 80 °C under reflux stirring. Then, a solution of 1.0, 2.0, 3.0 or 4.0 equivalent of QAS concentration with regards to the cation exchange capacity (CEC) dissolved in 20 mL of deionized water was added slowly to the Mt dispersion. The ion exchange reaction was carried out for 4 h at 80 °C under reflux, and a milky white flocculent precipitate was obtained by suction filtration. The precipitate was washed many times with deionized water until the filtrate was free of Cl^-^ ions (using 0.1 mol/L AgNO_3_ aqueous solution to detect Cl^−^ ions). The resulting products were dried in a vacuum oven at 65 °C for 24 h. Finally, the dried OMt were grounded through 200 meshes and sealed in a glass tube for use.

### Experimental characterizations

#### Fourier infrared transform spectroscopy

FT-IR of corresponding QAS, Mt, and OMt were obtained on a Thermo Scientific Nicolet iS10 infrared spectrometer. OTAC samples were characterized with the KBr pressed disk technigue (100 mg of KBr per 1 mg of sample). The wavelengths are measured from is 400 to 4000 cm^−1^. BDAC and TOMAC samples were characterized with the ATR method, and the wavelengths are measured from 650 to 4000 cm^−1^. The software package of Thermo Fisher is used for spectral analysis.

#### X-ray diffraction

XRD was used to characterize the change of interlayer space of OMt. XRD patterns were collected using Cu-Kα (λ = 0.154 nm) radiation on a Rigaku SmartLab X-ray diffractometer at a working voltage of 40 kV and a current of 30 mA, and the angular (2θ) range of 1°–10° at a scanning rate of 2°/min. The basal spacing of d_001_ was calculated according to the 2θ values of the 001 reflection on the XRD patterns using the Bragg equation.

#### Thermogravimetric analysis

The adsorption amount of QAS on Mt and the thermal stability of corresponding OMt were studied on a NETZSCH TA-Q500 TGA instrument. About 5 to 10 mg of sample was characterized from 25 to 795 °C under a nitrogen atmosphere with a heating rate of 10 °C/min.

### Molecular simulations

The Monte Carlo (MC) method was used to perform molecular simulations of the OTA-Mt, BDA-Mt, and TOMA-Mt model systems, to investigate the influence of the number of octyl chains and the loading of QAS on the Mt interlayer space. Attempts were made to reasonably understand the experimental characterizations by evolution of the conformation and energies of the intercalated QAS cations in interlayer space of the Mt.

#### Model building

Both Mt and pyrophyllite belong to the montmorillonite-saponite family and have the same aluminosilicate sheet structure^23^. Due to the lack of X-ray diffraction data of Mt crystals, we replaced the pyrophyllite crystal structure isomorphically^24,25^, that is, any Mg^2+^ ion in every four unit cells was replaced by an Al^3+^ ion, and then the system was neutralized with counterions of Na^+^ cations to obtain the Na-Mt crystal structure. The structural formula is Na_0.75_[Si_8_] (Al_3.25_Mg_0.75_)O_20_(OH)_4_, and the unit cell parameter was a = 0.5254 nm, b = 0.9085 nm, c = 1.0257 nm, α = γ = 90°, β = 99.82°^26^. The geometrical parameters of the three QAS cations of OTA^+^, BDA^+^, and TOMA^+^ are taken from quantum chemistry calculations by Gaussian program. The optimization method and basis set are B3lyp/6-31G.

The simulation system are composed of two Mt layers and a certain number of intercalated QAS cations. Among them, one Mt layer was composed of 6a × 4b × c Na-Mt crystal cells. To ensure the electrical neutrality of the simulation system, when a QAS cation is inserted, a Na^+^ cation is randomly removed in the system, and counterions of Na^+^ cations are evenly placed between and outside the Mt layers. In order to simplify the sampling, the QAS cations and Mt layers are rigid structures, and the influence of water molecules on the system is ignored.

It should be noted that in the following simulations only the all-trans alkyl tails conformation (staggered conformation) with the lowest molecular energy for the QAS ions be chosen. In order to illustrate the feasibility of conformation selection, an attempt was made to increase the proportion of QAS cations in gauche conformation in the simulation system. The structures and conformation information of the staggered and gauche QAS cations are shown in Fig. S1. The simulation results show that changing of the conformation and conformation ratio of the quaternary ammonium salt ions in the simulation system has little effect on the intercalation spacing of Mt. See Figs. S2 and S3 of Support Information for details.

#### Simulation methods

The MC simulation program used is developed on a self-made program^27^. The force field is a full atomic force field. The non-bond potential energy parameters (ε, σ) and electrostatic interaction parameters of each atom are shown in Table 1. In the simulation, the 2D periodic boundary condition is selected in the Mt layer, and the Z-axis direction perpendicular to the Mt layers, that is, the 001 direction of the crystal surface is aperiodic. The truncation radius of van der Waals interaction is 1.0 nm; The system is optimized by step scan with the stepsize of 0.01 nm, the scanning range between 1.20 and 2.70 nm. The random translation and rotation of the QAS cations as well as the random translation of sodium ions in the interlayer of the Mt constitute two Markov random walking chains, which forms a cycle, where each chain consists of 10,000 random walking steps. The simulation was run for 10 cycles to obtain an equilibrium conformation.

**Table 1:** Non-bond potential energy and charge parameters of Mt and QAS cations^25,28^.

Atom		*q*/(e)	*σ*/(Å)	*ε/*(Kcal/mol)
Na-Mt	Si	1.20	1.84	3.153
	Mg	2.00	0.00	0.00
	Al	3.00	0.00	0.00
	Na	1.00	2.586	0.10
	H	0.424	0.00	0.00
	O(t)^a^	− 1.00	3.166	0.156
	O(o)^b^	− 1.424	3.166	0.156
	O(a)^c^	− 0.80	3.166	0.156
OTA^+^/BDA^+^/TOMA^+^	N	0.00	3.25	0.170
	C1^d^	0.25	3.96	0.145
	C2^e^	− 0.12	3.50	0.105
	C3^f^	− 0.18	3.75	0.105
	H	0.06	2.50	0.030

^a^O (t) represents tetrahedral oxygen.

^b^O (o) represents octahedral oxygen.

^c^O (a) represents terminal oxygen (apical), that is, structural hydroxyl oxygen.

^d^C1 is the carbon connected to N^+^.

^e^C2 is the carbon in CH_2_.

^f^C3 is the carbon in CH_3_.

## Results and discussion

### FT-IR spectra of QAS, Mt and OMt

**Figure 1:**
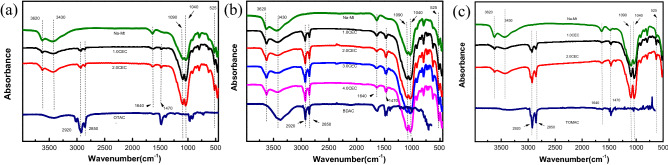
FT-IR spectra of Na-Mt, QAS, and Mt modified with the QAS at different CEC concentration: (**a**) OTA-Mt, (**b**) BDA-Mt, (**c**) TOMA-Mt.

The infrared spectra of the studied samples are shown in Fig. 1a–c. We focus on the changes of some characteristic peaks of the clay before and after organic modification, including the stretching vibrations of free water within the Mt interlayer, the stretching vibrations of alkyl group of the intercalated QAS cations, and the vibrations of Al–O–Al and Si–O-Si in aluminosilicate sheets. First, for all modified samples, the absorption peaks around 3430 cm^−1^ and 1640 cm^−1^ attributed to asymmetry and symmetric stretching of water within the OMt interlayer show a significant decrease in intensity comparing with those of Na-Mt, suggesting that the hydrophobicity of OMt is enhanced due to the intercalation of organic QAS cations. The related reduction of water content in OMt layers is discussed further in TG analysis in the next section. Second, new peaks near 2920 cm^−1^ and 2850 cm^−1^ assigned to the asymmetrical and symmetric stretching of -CH_2_ appear in all OMt complexes^4,18^, indicating that QAS cations have entered the Mt interlayer. The intensity of those peaks for BDA-Mt and TOMA-Mt are significantly larger than that of the corresponding OTA-Mt, because the former contains more alkyl groups. In addition, when the concentration of QAS of the preparation solution increases from 1.0 CEC to 2.0 CEC, the peak intensity of the BDA-Mt and TOMA-Mt shows a significant increase, suggesting that the loading of QAS cations in the OMt interlayer has a positive correlation with its concentration. Third, the vibration peaks around 1090 cm^−1^ and 1040 cm^−1^ attributed to asymmetric stretching of Al–O–Al and Si–O–Si of Mt layers have no obvious changes in intensity and displacement, which implies that the intercalation of QAS has little effect on the structure of raw Mt layers.

It should be noted that because the QAS loading has reached saturation at 1.0 CEC of OTA-Mt and 2.0 CEC of TOMA-Mt, respectively, we do not show the infrared spectrum corresponding to higher concentrations in Fig. 1.

### TG analysis

**Figure 2:**
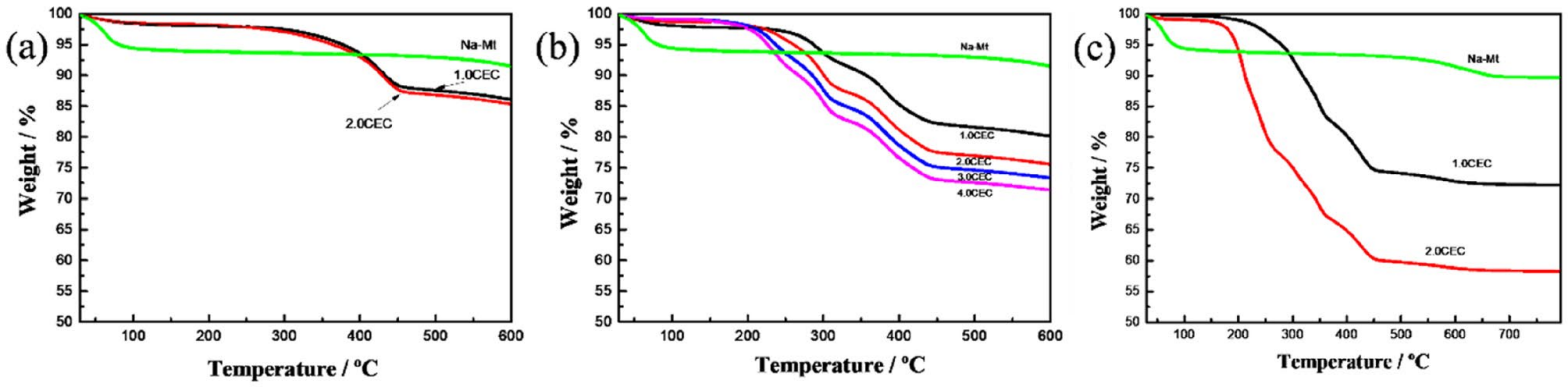
TG curves of Na-Mt and Mt modified with various QAS concentration.

In order to investigate the loading of QAS cations between the Mt layers and its thermal stability information, we performed TG analysis of three modified OMt complexes with various concentration of QAS (Fig. 2). For all three complexes, the TG curves show that as the heating temperature rises, the mass loss of the three complexes can be roughly divided into three stages: dehydration of physically adsorbed water of the OMt (0–100 °C); thermal decomposition of the intercalated QAS cations (200–450 °C), and removal of hydroxyl groups from aluminosilicate sheets (450–700 °C).

It should be noted that the TG curve of 1.0 CEC OTA-Mt complex almost overlaps with that of 2.0 CEC OTA-Mt complex, while the TG curve of 2.0 CEC TOMA-Mt almost overlaps with that of 3.0 CEC TOMA-Mt, suggesting that the loading of QAS cations have reached saturation at 1.0 CEC OTA-Mt and 2.0 CEC TOMA-Mt complexes, respectively, and it no longer changes with the increase of the concentration of the QAS. This is further verified by the XRD analysis. Therefore, in the following sections we only discuss the OTA-Mt and TOMA-Mt complexes with the QAS concentration up to 1.0 CEC and 2.0 CEC, respectively.

From the TG curves, it is found that the mass loss of water for three OMt complexes decreases significantly comparing with that of raw Mt, furthermore, the water content decreases with increasing the number of octyl chains. For example, the mass loss of water is only within 1% for the OMt comparing to 7% for raw Mt. The reduction of the water between OMt layers indicates that intercalation of QAS cations will change raw Mt from hydrophilic to hydrophobic, and this transition occurs more easily for the OMt modified by the QAS with many octyl chains.

**Figure 3:**
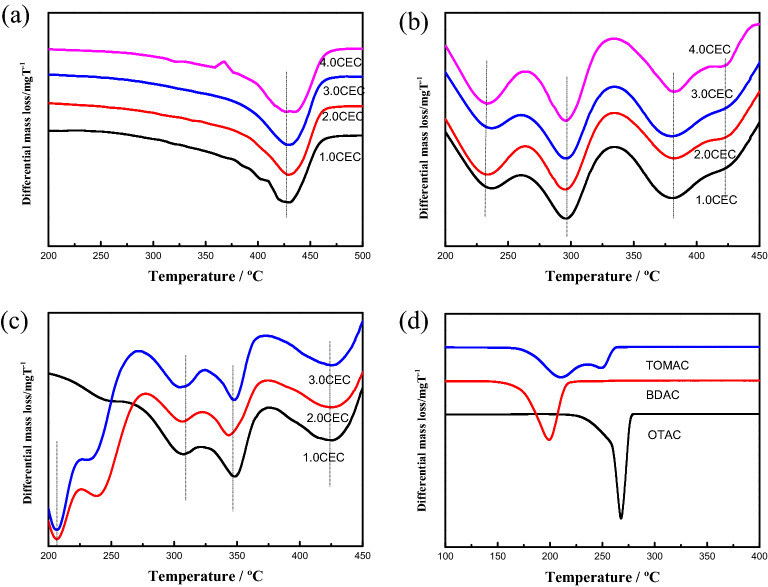
DTG curves of QAS and Mt modified with various QAS concentration: (**a**) OTA-Mt, (**b**) BDA-Mt, (**c**) TOMA-Mt, and (**d**) the QAS.

We pay more attention to the second stage of mass loss for QAS in Fig. 2. It can be seen that the initial thermal decomposition temperature of the intercalated QAS cations increases significantly comparing with that of the QAS in bulk phase, which indicates that thermal stability of the QAS cations is enhanced under the action of the Mt layers; the thermal decomposition temperature of the OTA-Mt complex is the highest, followed by the BDA-Mt complex, and the TOMA-Mt complex is the lowest. This is because the more the octyl chains, the lower the thermal stability of the QAS cations. In addition, with the increase of QAS concentration, the mass loss of the intercalated cations for all three complexes increase, indicating that there is a positive correlation between the QAS concentration and the loading of intercalation cations. It is worth noting that for the OMt complexes modified by the QAS with many octyl chains, i.e., the BDA-Mt and TOMA-Mt complexes, the intercalated QAS cations, BDA^+^, TOMA^+^, exhibit a wave-like downward TG curves. In order to better understand the thermal decomposition process of the intercalated QAS cations, the differential thermogravimetric curves (DTG) of the three complexes is given in Fig. 3.

It shows in Fig. 3 that for the OTA-Mt complex, the curve of mass loss of intercalated cations has only one peak, and the maximum mass loss rate is at 430 °C. However, for the BDA-Mt and TOMA-Mt complexes, the thermal decomposition process of intercalated cations becomes complicated. For the BDA-Mt complex, the cations decomposition can be divided into 3 distinct stages, and the corresponding maximum mass loss rates are at 230, 290, and 380 °C, respectively. Although a shoulder is present at 425 °C, it is weak. For the TOMA-Mt complex, the situation is more complicated. The concentration of QAS has a great correlation with its thermal decomposition process. For the 1.0 CEC TOMA-Mt complexes, there are also three obvious decomposition stages for TOMA^+^ ions, corresponding maximum mass loss rates are at 310, 345, and 420 °C, respectively. With the increase of the concentration of TOMAC, a new mass loss peak appears in the low temperature region near 200 °C, which obviously corresponds to the decomposition process of TOMAC in bulk phase.

In order to compare the thermal stability of the QAS before and after intercalation easily, Fig. 3d also shows the DTG curves of the three kinds of QAS (OTAC, BDAC, and TOMAC) in bulk phase. It can be seen that the maximum mass loss rates of the three QAS before intercalation are at 260, 200, 230–50 °C, respectively. And the main mass loss peaks of the three intercalated QAS cations appear in a wide temperature range from 200 to 450 °C, indicating that the thermal stability of the intercalated cations changed significantly. In fact, except for a part of intercalated TOMA^+^ cations, which still maintains the thermal stability of bulk phase, the thermal stability of intercalated QAS cations has been significantly enhanced.

The appearance of multiple mass loss peaks during the thermal decomposition of intercalated QAS cations implies that there exist several possible configurations of intercalated cations between the Mt layers. The related discussion will be given later in the section of MC simulations.

### XRD diffraction

**Figure 4:**
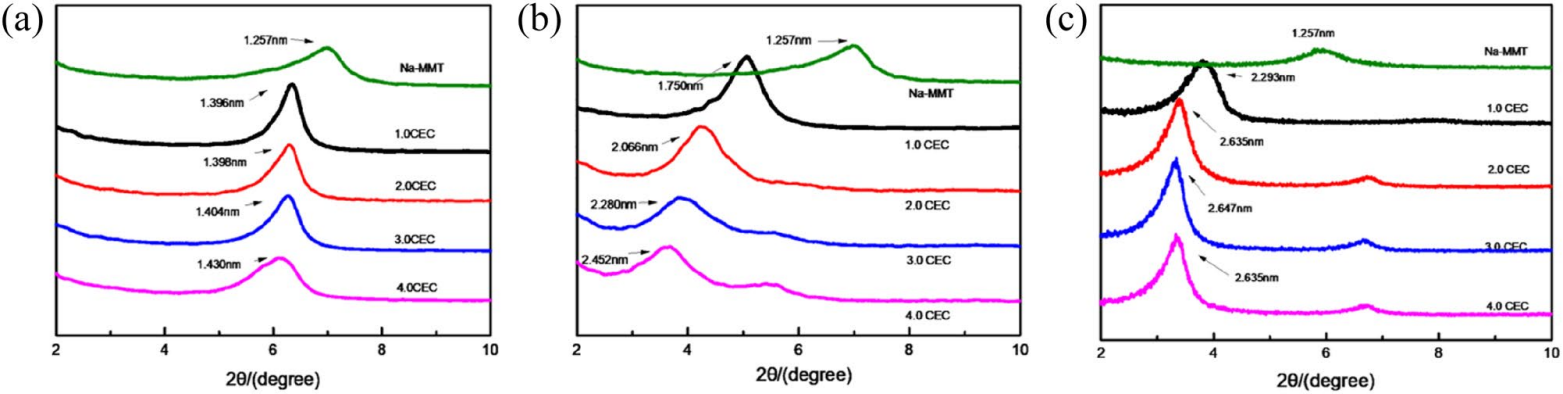
XRD patterns of Na-Mt and Mt modified with various QAS concentration: (**a**) OTA-Mt, (**b**) BDA-Mt, (**c**) TOMA-Mt.

In order to investigate the effect of different QAS loading on the interlayer space of OMt, the observed basal spacing of the studied complexes was measured by X-ray diffraction, and the d_001_-value was calculated according to the Bragg equation: λ = 2d_001_sinθ, where λ and θ are incident wavelength and angle between incident X-ray and crystal plane (001), respectively^29^. The XRD patterns of the Mt and OMt are shown in Fig. 4. As can be seen from Fig. 4, with the increase of the QAS concentration, the d_001_-value shows an increasing trend. For mono octyl chain OTA-Mt complex, the increase of d_001_ is not significant. However, for dioctyl chains BDA-Mt complex, the interlayer space changes significantly, and d_001_ increases from 1.750 nm at 1.0 CEC to 2.452 nm at 4.0 CEC (a significant increase over 0.7 nm). For trioctyl chains TOMA-Mt complex, when TOMAC concentration increases from 1.0 CEC to 2.0 CEC, the interlayer space increases from 2.293 to 2.635 nm, but for samples with larger QAS concentration, the d_001_-value no longer increases. The change of the basal spacing of investigated OMt indicates that the effect of increasing QAS concentration on the interlayer space is different, and the change trend is consistent with the TG analysis. In general, as the number of QAS octyl chain increases, the interlayer space of the OMt increases, i.e., TOMAC has a larger effect on the expansion of the interlayer space of Mt than BDAC, and BDAC larger than OTAC.

In addition, as the QAS loading increases, the XRD patterns of the OMt complexes modified by QAS with many octyl chains showed a double diffraction peak. The patterns suggest that the weak peak for the TOMA-Mt complex at about 6.67° can be attributed to the 002 reflection, indicating that as the amount of intercalation increases, the OMt complexes becomes more crystallized. A weak diffraction peak appears at 5.60° in the XRD diagram for the OMt complex, and the peak intensity becomes more prominent with the increase of BDAC concentration. Interestingly, this diffraction peak cannot be attributed to the 002 diffraction peak. We speculate that there may be two stable configuration of BDA^+^ cations in the Mt interlayer, which resulted in different expansion effect^30^. In order to better explain the reason for the change of OMt interlayer space, an analysis of MC simulations is given in the following section.

### MC simulations

**Figure 5:**
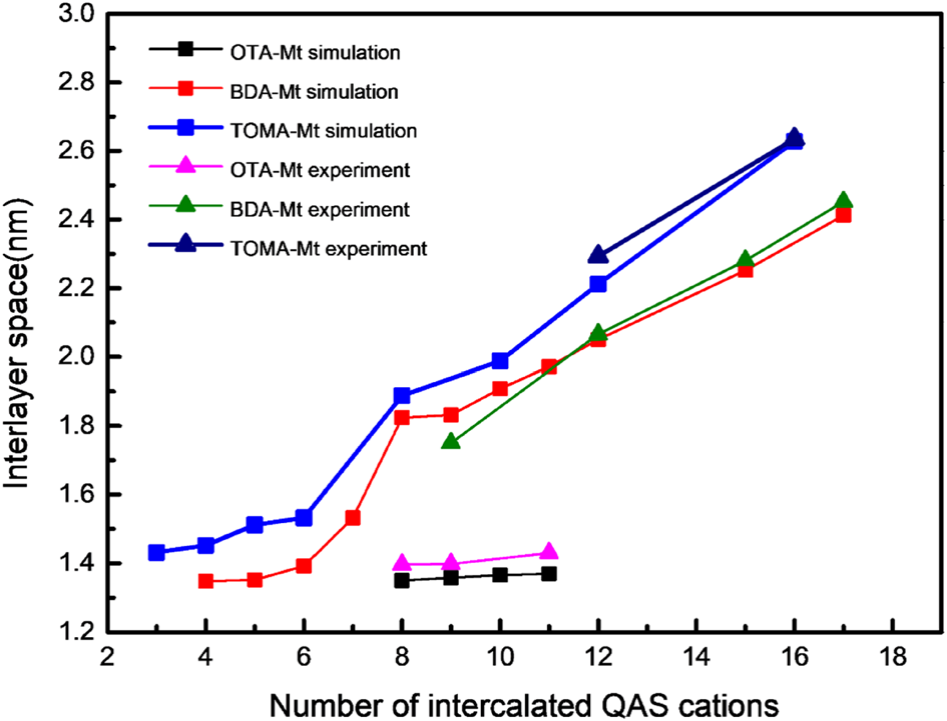
Computational and Experimental relationship between the number of intercalated QAS cations and the interlayer space of the three complexes.

Within the framework of MC simulations, the equilibrium conformation of the three OMt complexes with different loading of intercalation agents was optimized by scanning the distance of OMt interlayer space. The calculated d_001_ as well as those obtained by XRD characterization are shown in Fig. 5. It should be noted that in order to easy to comparison, the CEC concentration of the QAS in the experiment has been converted to the number of intercalated QAS cations through TG characterization data (mass loss of the QAS) to build the simulation system. It can be seen from Fig. 5 that when the QAS loading is the same, the d_001_ obtained by MC simulations agree well with those by the XRD analyses for the three complexes, which implies that the model and force field parameters selected in MC simulations are credible, and it also lays a solid foundation for the evaluation of the simulation results below.

In order to easily distinguish the distribution of QAS cations between the Mt layers, we simply divided the interlayer space into surface and inner areas according to the position of QAS cation to aluminosilicate sheets. Thus, the QAS cations distributions are divided into three cases, namely the surface area, the inner area, and the cross area, where the QAS cation is in the surface area, the inner area, and both the surface and inner area, respectively.

**Figure 6:**
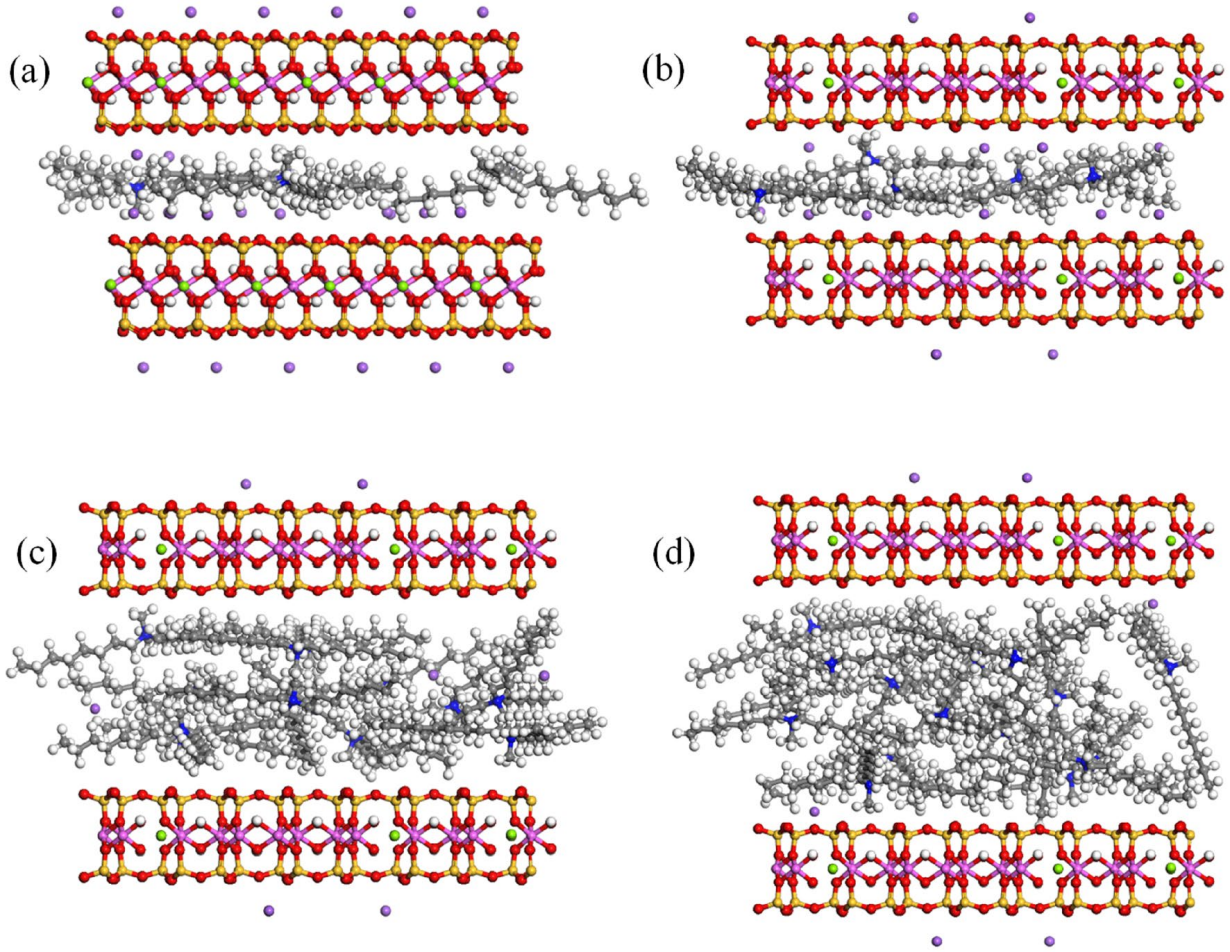
Snapshots of the simulated equilibrium of TOMA-Mt complexes with various intercalation: (**a**) 4 TOMA^+^ cations, (**b**) 5 TOMA^+^ cations, (**c**) 12 TOMA^+^ cations, (**d**) 16 TOMA^+^ cations.

According to the QAS position statistical analyses, it is found that for the OTA-Mt complex, the QAS cations exist only in the surface area. When the number of intercalated QAS cations is between 8 and 11, OTA^+^ exhibits a single layer distribution. As the loading of salt cations increases, its distribution density between the Mt layers increases, but it still maintains a single-layered distribution, so it has little effect on the interlayer space. Because experiments have shown that the interlayer space of OTA-Mt complex does not change at high QAS concentrations, we have not carried out MC simulations on the complexes with higher loading QAS cations. For the BDA-Mt complex, when the number of intercalated cations is from 4 to 6, BDA^+^ cations show a single-layered distribution, and when the number increases to 8–9, BDA^+^ cations show a double-layered distribution (Fig. 5). The distribution of BDA^+^ cations transition from single-layered to double-layered distribution resulted in a stepwise increase of the d_001_-value. Unlike the OTA-Mt complexes that salt cations exist only in the surface area, some BDA^+^ cations begin to appear in the inner area when the BDA^+^ loading increases, resulted in the interlayer space increases linearly. A similar trend also appears for the TOMA-Mt complexes. However, the change of d_001_-value for TOMA-Mt is not with a stepwise when the loading of TOMA^+^ cations increases as that of the BDA-Mt complexes, due to the complexity of the structure of TOMA^+^ cation. In fact, as the loading increasing, BDA^+^, TOMA^+^ cations are not only positioned in the surface area in a horizontal direction, but also exist in an inclined manner in the inner area and the cross area. As a result, these QAS cations are distributed not only in simple integer layers, but also in non-integer layers. Snapshots of the equilibrium configuration of the TOMA-Mt complex with various loading are shown in Fig. 6. Generally speaking, the effects of dioctyl and trioctyl chain QAS on interlayer space of OMt are significantly stronger than that of mono octyl chain QAS. This is because the QAS with many octyl chains can arrange in a multi-layered distribution in the OMt interlayer.

Using the simplified distribution model of QAS cations described above, we attempt to qualitatively explain the DTG curves of the intercalated cations during the thermal decomposition process. For mono octyl chain OTA-Mt complexes, since the intercalated OTA^+^ cations exist only in the surface area and can be attributed to one distribution pattern, the DTG curve has only one peak of mass loss. At the same time, the OTA^+^ cations interact with aluminosilicate sheet through a strong ionic bond, thereby significantly improving the thermal stability of the intercalated cations, causing its mass loss peak to move to a high temperature region. For dioctyl and trioctyl chain complexes, when the QAS concentration is above 1.0 CEC, the intercalated cations shows a multi-layered distribution, which are not only distributed in the surface area but also in the inner area and the cross area, attributed to 3–4 distribution patterns, resulting in a DTG curve with 3–4 mass loss peaks. For 2.0 CEC TOMA-Mt complex, the intercalated TOMA^+^ cations are distributed in all three areas. In particular, those cations in the inner area is far away from Mt layers, thus, the interaction of the cations with aluminosilicate sheets is small. Therefore, thermal decomposition of those cations behave like the bulk phase of TOMA^+^ cations, and a significant mass loss peak appears in 200 °C region.

**Figure 7:**
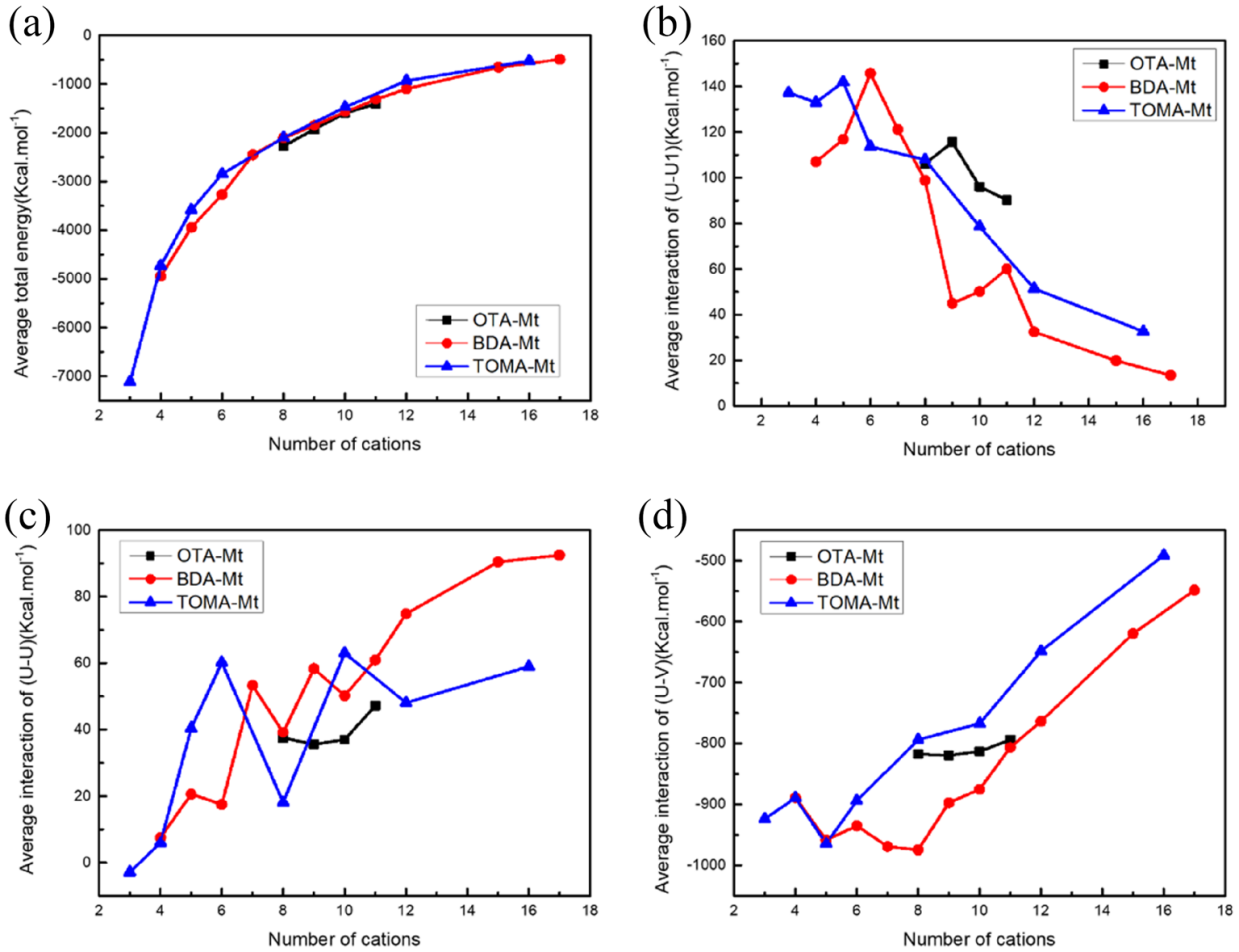
The variations of the average total energy of the system (**a**), the average interaction energy between the QAS cations with the number of sodium ion (**b**), with Mt layers (**c**), and with the other QAS cations (**d**), respectively, as the increase of the loading cations.

In the following, an energy analysis is used to explain the distributions of the QAS cations in the OMt interlayer and the change of the basal spacing of the OMt complexes. It can be seen from Fig. 7a–d that the trends of the total energy of three investigated complexes are consistent, and intercalation of the QAS cations makes the total energy of the system to be negative. However, with the increase of the loading cations, the total energy gradually increases and tends to a horizontal platform, indicating that the thermal driving force of intercalation gradually decreases, and the amount of intercalation will trend to be saturated. In addition, it can be seen from decomposition of the total energy that the average interaction of intercalated QAS cations with Mt layers(U-V) is the main contributors to the total energy, and the average interaction of intercalated QAS cations with sodium ions(U-U1) and other QAS cations(U-U) have a smaller contribution.

For the three OMt complexes, the average interaction energy of the QAS cations is large when the loading of cations is small, and it does not change much with the number of the intercalated cations. This is consistent with the fact that with a small loading the QAS cations only exist in the surface area. However, when the loading of cations is large enough, the surface area is full of the intercalated cations, and the cations will be distributed to the cross and inner areas, away from Mt layers, resulting in a gradual increase in average interaction energy.

Interestingly, as can be seen in Fig. 7b,c, for the BDA-Mt and TOMA-Mt complex, the average interaction energy between the QAS cations with sodium ion (b), with Mt layers (c) showed a zigzag trend with the increasing number of cations. Further observations show that these energy fluctuations are basically consistent with the changes of the distribution pattern of QAS cations between the Mt layers. Taking the curve of the average interaction energy between QAS cations for the TOMA-Mt complex as an example, it is easy to find that when the intercalated QAS cations are few, they are in a single-layered distribution pattern, increasing the number of cations, the repulsion increases with a increase of the average interaction energy, while when they transition from one pattern to another pattern(i.e., from single-layered to double-layered distribution), due to the enlarging of the interlayer space, the distance between cations increases and the repulsion decreases, resulting in a decrease of the interaction energy. This situation repeats as the cation distribution pattern changes with the increasing number of cations, resulting in a zigzag curve of the interaction energy.

## Conclusions

FT-IR, XRD, and TG characterization showed that three kind of QAS cations investigated were inserted into the interlayer of Na-Mt. A comparison of the basal spacing of OMt complexes with that of raw Na-Mt suggested that intercalation will enlarge the interlayer space of OMt complexes. The increase of the number of octyl chains of intercalated QAS helps the expansion of OMt interlayer space. The increase of OTAC concentration has little effect on the change of the OMt interlayer space. The increase of BDAC and TOMAC concentration will continuously increase the interlayer space, and the expanding effect of the TOMAC is obvious. However, when the TOMAC concentration is 2.0 CEC, the expanding effect reaches saturation. In addition, the organic modification of the QAS results in the modified Mt changing from hydrophilic to hydrophobic.

The MC simulations of the distributions of intercalated cations in the Mt interlayer space explain experimental results. According to MC simulations, the changes of the basal spacing of the OMt complexes by three kinds of intercalated QAS cations with various loading were obtained. As the loading cations increases, the distribution of OTA^+^ cations in the interlayer is mono-layered pattern, BDA^+^ cations change from mono-layered to double-layered pattern, and TOMA^+^ cations change from mono-layered to multi-layered pattern, which explained the experimental characterizations. From the change of the average energy of the intercalated cations, it was found that the interaction of the QAS cations with the Mt layers and the sodium ions is the main contribution to the total energy.

In recent years, the organic modification of Mt has been widely used in the field of water treatment. Our group is now exploring the adsorption experiments of phenolic compounds in water. The original results showed that the adsorption performance of modified montmorillonite for phenolic compounds was significantly improved. We expect that the modification of Mt by QAS cations can provide a theoretical basis for the preparation of composites with excellent properties in industrial application in the future.

## Supplementary Information

Supplementary Figures.

## Data Availability

All data generated or analysed during this study are included in this published article (and its Supplementary Information files).
