# Supplementary material for: Experimental and modeling investigation of organic modified montmorillonite with octyl quaternary ammonium salt
Source: Sci Rep. 2022 Aug 22;12:14305. doi: 10.1038/s41598-022-18253-1 (PMC9395535; doi:10.1038/s41598-022-18253-1)
Supplement: Supplementary file 1 — Supplementary Figures. [file 41598_2022_18253_MOESM1_ESM.docx]

**SUPPLEMENTARY INFAOMATION FOR**

Experimental and Modeling Investigation of Organic Modified Montmorillonite with Octyl Quaternary Ammonium Salt

Hongyan Liu^a^, Chengxin Guo^a^, Yingna Cui ^a,b*^, Jingmei Yin ^a,b^and Shenmin Li^a,b*^

^a^College of Environment and Chemical Engineering, Dalian University, Dalian, 116622, China

^b^Liaoning Key Laboratory of Bioorganic Chemistry, Dalian University, Dalian, 116622, China

Corresponding Author: e-mail:lishenmin@dlu.edu.cn.

ORCID:0000-0002-9518-7247


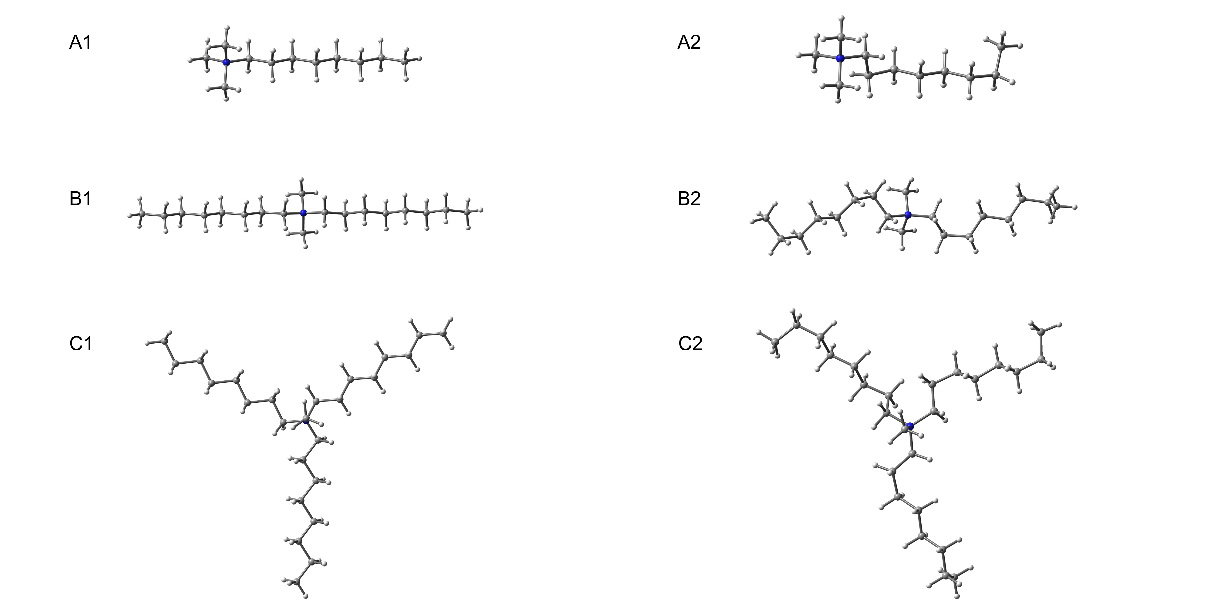


**Figure. S-1** A1 The structures and conformation information of the staggered OTA^+^. A2 The structures and conformation information of the gauche OTA^+^. B1 The structures and conformation information of the staggered BDA^+^. B2 The structures and conformation information of the gauche BDA^+^. C1 The structures and conformation information of the staggered TOMA^+^. C2 The structures and conformation information of the gauche TOMA^+^.


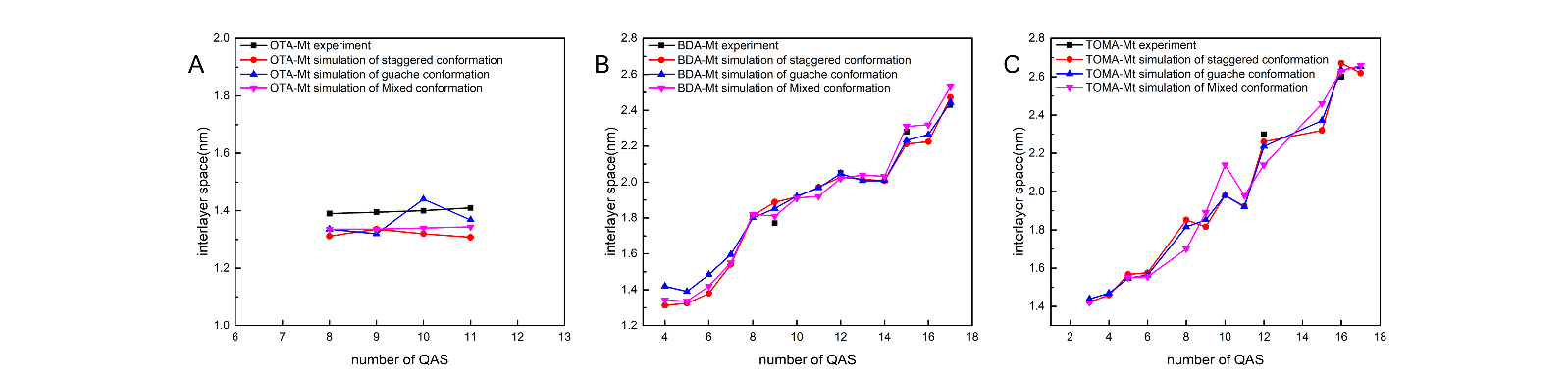


**Figure. S-2** Computational and Experimental interlayer space of the three complexes with variation of the conformation and number of intercalated QAS cations.


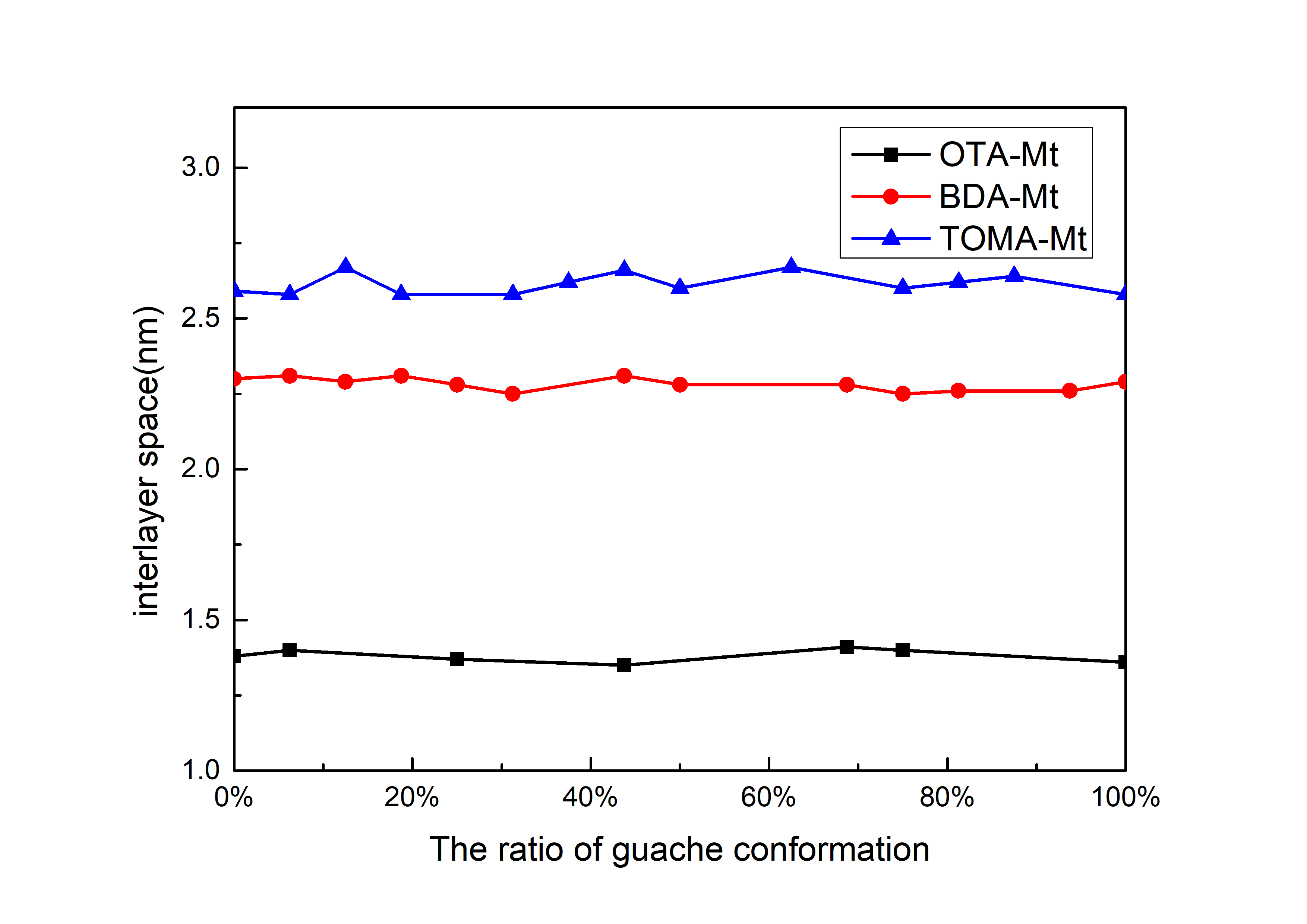


**Figure. S-3** Computational interlayer space of the three complexes with variation of the gauche conformation for the systems of 16 intercalated QAS cations.
